# Interaction of climate and socio-ecological environment drives the dengue outbreak in epidemic region of China

**DOI:** 10.1371/journal.pntd.0009761

**Published:** 2021-10-04

**Authors:** Chenlu Li, Xiaoxu Wu, Scott Sheridan, Jay Lee, Xiaofeng Wang, Jie Yin, Jiatong Han

**Affiliations:** 1 State Key Laboratory of Remote Sensing Science, College of Global Change and Earth System Science, Beijing Normal University, Beijing, China; 2 Southern Marine Science and Engineering Guangdong Laboratory, Guangzhou, China; 3 Department of Geography, Kent State University, Kent, Ohio, United States of America; 4 College of Environment and Planning, Henan University, Kaifeng, China; 5 Center for Disease Surveillance and Information Services, Chinese Center for Disease Control and Prevention, Beijing, China; University of Heidelberg, GERMANY

## Abstract

Transmission of dengue virus is a complex process with interactions between virus, mosquitoes and humans, influenced by multiple factors simultaneously. Studies have examined the impact of climate or socio-ecological factors on dengue, or only analyzed the individual effects of each single factor on dengue transmission. However, little research has addressed the interactive effects by multiple factors on dengue incidence. This study uses the geographical detector method to investigate the interactive effect of climate and socio-ecological factors on dengue incidence from two perspectives: over a long-time series and during outbreak periods; and surmised on the possibility of dengue outbreaks in the future. Results suggest that the temperature plays a dominant role in the long-time series of dengue transmission, while socio-ecological factors have great explanatory power for dengue outbreaks. The interactive effect of any two factors is greater than the impact of single factor on dengue transmission, and the interactions of pairs of climate and socio-ecological factors have more significant impact on dengue. Increasing temperature and surge in travel could cause dengue outbreaks in the future. Based on these results, three recommendations are offered regarding the prevention of dengue outbreaks: mitigating the urban heat island effect, adjusting the time and frequency of vector control intervention, and providing targeted health education to travelers at the border points. This study hopes to provide meaningful clues and a scientific basis for policymakers regarding effective interventions against dengue transmission, even during outbreaks.

## Author summary

Dengue is among the rapidly spreading vector-borne diseases and is widespread in tropical and subtropical areas. In China, dengue is a serious public health problem, especially in the southeastern regions with rapid urbanization, such as Guangzhou City. Since 1998, more than 40,000 dengue cases have been reported in Guangzhou. Dengue transmission is affected by multiple factors, including climate and different socio-ecological factors. This study indicates that temperature has a significant effect on the long-time series of dengue transmission, while socio-ecological factors play an important role in dengue outbreaks. The interactive effect of temperature and socio-ecological factors contributes to dengue outbreak in Guangzhou. Increasing temperature and surge in travel could cause a dengue outbreak in the future. We have made suggestions about policies and measures regarding its prevention in Guangzhou.

## Introduction

Dengue, as the most prevalent and rapidly spreading mosquito-borne infectious disease, is caused by four dengue viruses and is transmitted to humans via *Aedes* mosquitoes [1]. The disease is characterized by fever, headache, arthralgia and myalgia [2]. Dengue is spreading in more than 100 countries globally, mostly in the tropical and subtropical regions [3, 4]. Dengue is the deadliest mosquito-borne disease after malaria [4], with nearly 4 billion people at risk and over 20,000 deaths yearly [5]. In the literature prior to 2017, 291,964 cases of outbreak-related dengue were reported, most of which occurred in the Western Pacific region (72.4%), followed by the Americas (19.4%) [6]. Latin America has experienced the most severe dengue epidemic in history, with the total number of cases more than 1.3 times the number recorded in 2015 [7]. According to a report from the World Health Organization, dengue is one of the top ten global health threats in 2019 [8].

China is one of the most affected countries, accounting for 27.9% of all dengue cases from literature reviews [6]. In China, the earliest recorded dengue case was in the Yangze River Basin in the 19th century [9]. The first outbreak of dengue was reported in 1978 in Shiwan Town, Foshan City, Guangdong Province [10]. Since then, dengue epidemics have shown an increased trend in severity, and a spread towards the north [11]. In 2014, the unprecedented outbreak of dengue in Guangdong Province accounted for over 40,000 cases, which exceeded the historical average by two orders of magnitude and was far beyond the epidemic threshold [12].

Dengue transmission is affected by multiple factors, including climate change and different socio-ecological factors due to the intrinsic complexity of its transmission being related to virus-vector-human interactions [13, 14]. Climate and its variability in temperature [15], precipitation [16], humidity [17, 18], wind velocity [19], vapor pressure [20] and extreme weather events [20] can impact dengue transmission in terms of three essential aspects: the dengue virus itself, *Aedes* mosquitoes, and the transmission environment [11, 21]. Socio-ecological factors, such as gross domestic product (GDP) [22], urbanization [23], travel [24], bodies of water [25] and vegetation [22], are also associated with dengue incidence. Therefore, research on the relationship between climate as well as socio-ecological factors and dengue transmission has become an active area.

First, since dengue is sensitive to climate conditions, many studies have explored the impact of climate on dengue transmission. One way this has been tackled is by analyzing the correlation between climate factors and dengue incidence [26]. For example, Lu et al. [27] indicated that minimum humidity and minimum temperature were both positively associated with the dengue incidence at a lag of 1 month, whereas wind velocity was negatively associated with dengue over the same period. Additionally, early warning systems of dengue outbreaks have also been established based on climate factors [28–32].

Second, some studies investigated the effect of socio-ecological factors on dengue transmission [33–36]. A study in Guangzhou City during 2012–2017 used a geographically weighted regression model to examine the relationships between dengue epidemics and GDP, the traffic system (road density, bus and/or subway stations), and urban villages, and found urban villages likely acted as special transfer stations for dengue epidemic [37].

Third, a few studies have combined climate and socio-ecological factors to explore the effect of each factor on dengue transmission. One study applied a new modeling framework based on compartmental systems to analyze the impacts of mosquito control, human mobility and temperature on dengue transmission [14].

Abundant studies in recent years have focused either on examining the impact of climate or socio-ecological factors on dengue, or only analyzed the individual effects of a single factor on dengue transmission [13, 38]. It is noted that dengue transmission is based on the interaction of multiple factors [14, 39]. Analyzing the interactive effects of climate and socio-ecological factors on dengue, and identifying the dominant factors causing dengue outbreaks have important guiding significance for local dengue prevention and control. However, studies on the interaction of both climate and socio-ecological factors on dengue incidence are rare. This study uses the geographical detector method to explore the interaction of these key factors on dengue transmission, based on the long-term data and outbreak data in Guangzhou, China, identifies the main factor of dengue outbreak and assesses the possibility of dengue outbreaks in the future.

## Methods

### Study area

**Fig 1:**
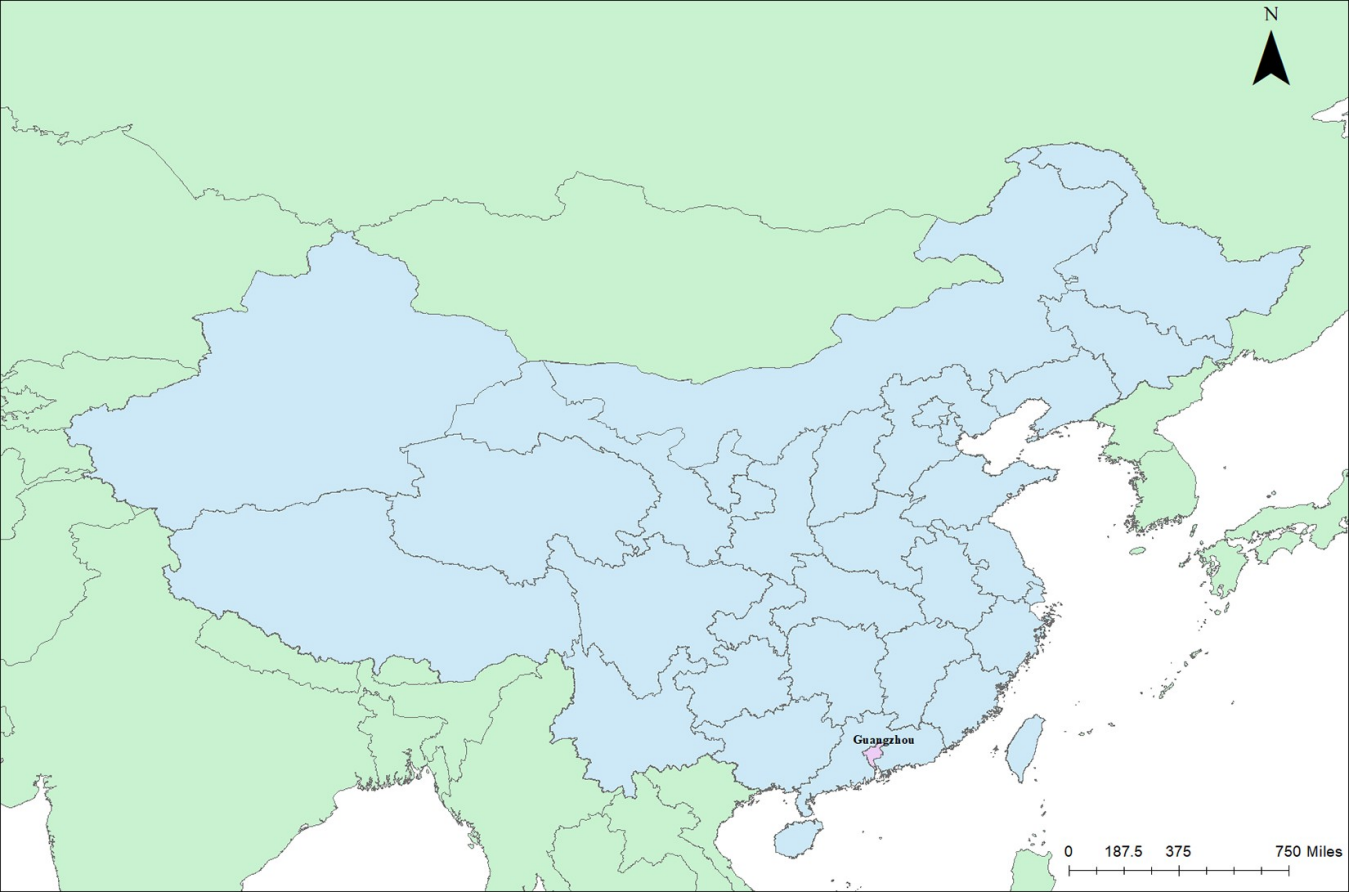
The location of study area in China. The base layer of the map is from Natural Earth (https://www.naturalearthdata.com/downloads/10m-cultural-vectors/).

Guangzhou, the capital city of Guangdong Province, is located in southern region of China (22°26′–23°56′N, 112°57′–114°3′E) (Fig 1). Guangzhou was chosen as the study area for three reasons. First, Guangzhou features a subtropical climate with adequate sunshine and heat [31]. The annual mean temperature, rainfall and relative humidity in this city are about 22°C, 1846mm and 76%, respectively. The climate is characterized as warm and rainy, which provides a suitable environment for the survival, growth and breeding of *Aedes* mosquitoes, vectors of dengue transmission [31, 40, 41]. Second, Guangzhou belongs to the Pearl River Delta Economic Zone, which is one of the highly urbanized and densely populated regions in the world. Rapid urbanization [37] and frequent population movement [24] make Guangzhou a high-risk area for dengue epidemics. Third, Guangzhou, as an endemic region, reported dengue cases almost every year over the last 30 years. Especially in 2014, it experienced the most serious outbreak of dengue on record with over 40,000 cases [42], which accounted for 79.32% of the total number of cases in 2014 in mainland China [35].

### Data collection

#### Disease data

In China, each case of a notifiable disease (including dengue) must be reported to the real-time and online National Infectious Diseases Monitoring Information System Database maintained by the Chinese Center for Disease Control and Prevention (CCDC). In this study, we collected monthly data for dengue cases in Guangzhou between January 1998 and December 2014 from CCDC. Collected dengue cases were all clinical or laboratory confirmed by hospital diagnosis and reported to the CCDC.

#### Climate data

Monthly climate data for Guangzhou during January 1998 and December 2016 were retrieved from the China Meteorological Data Sharing Service System. The data between January 1998 and December 2014 were used for the historical analysis, while monthly data during 2015–2016 were used to do predictive assessment. Our previous research [31] determined three climate factors as key factors and thus were also considered in this study. These factors are monthly mean temperature (MeanT), monthly mean cumulative precipitation (Precipitation), and monthly mean relative humidity (MeanRh).

#### Socio-ecological data

We collected annual data during 1998–2016 on thirteen factors involving mainly socio-ecological variables that have been suggested to be associated with dengue incidence in Guangzhou [43]. These variables cover a spectrum of different aspects, namely the population characteristics, population mobility, urbanization and vector habitat. Four key factors were selected from initial thirteen factors based on our previous research [43], and were eventually used in this study. They were population density (Pop_Den), night-time light, the number of domestic and overseas travelers (Travel), and proportional area of forest and grassland and waters (Landuse). The time period and the respective use of socio-ecological data were the same as that of the climate data.

#### Projection data

Projected climate data were derived from three earth system models including IPSL-CM5AMR [44], MIROC-ESM [45] and BNU-ESM [46]. The three models performed well for simulating temperature in China. They had been proven to be excellent in predicting the occurrence of infectious diseases [31, 47]. A set of emission scenarios referred to as Representative Concentration Pathways (RCPs) reflect various possible combinations of economic, demographic, technological, and policy developments [48]. We used the outputs from 2017 to 2050 under the RCP 4.5 scenario in this study, since this scenario is the secondary emission scenario, a stabilization scenario [49].

We employed the bilinear interpolation to downscale the data from globe-scale models to a city level [47]. In addition, we used an equally weighted ensemble of the three models to obtain an ensemble-mean estimate of climate data, in order to avoid initial condition, boundary condition, parameter, and structural uncertainties [50].

Regarding population projection, a report projected the population for the world’s largest cities in the 21^st^ century based on four scenarios including World Urbanization Prospects (WUP) and three basic “shared socioeconomic pathways” (SSP1, SSP2, and SSP3) proposed by the US National Center for Atmospheric Research [51]. SSP1 is a sustainable scenario, where the world has made relatively good progress in sustainable development and has a high level of urbanization. SSP2 is the middle scenario, which assumes that urbanization is in the current trend. SSP3 is a fragmented scenario, characterized by a slow urbanization [52]. Based on the report, we extracted the future population data in Guangzhou from 2025 to 2050 under four models (i.e. SSP1, SSP2, SSP3 and WUP).

Night-time light is a useful index reflecting urbanization dynamics since it focuses more on the intensity of anthropogenic activity involving demographic dynamics, socioeconomic growth, and levels of urbanization [53]. Since there is currently no specifically predicted data on night-time light, its future trends were described based on analysis and projection of urbanization.

The development trend of Travel is similar to that of the tourism industry [54]. Since there was no predicted data for Travel, we used the trend of the tourism industry to describe the future trend of Travel. Analysis of urbanization and tourism industry was derived from related literature and government reports [55–57].

### Data analysis

We examined the effect and interaction of climate and socio-ecological factors on dengue transmission from two aspects, i.e. the long-time series of dengue cases (January 1998-December 2014) and the outbreak periods. With respect to dengue outbreaks, there is not yet any ubiquitously accepted definition [58, 59]. In this study, the dengue outbreak was defined as a month when the monthly number of dengue cases was above the threshold of the 3^rd^ quartile of all dengue cases. Based on this definition, we selected the monthly dengue cases of outbreak periods and corresponding climate and socio-ecological factors to do the analysis.

The geographical detector method [60] was used to investigate the spatial stratified heterogeneity of variables and tested the determinant power of driving factors accountable for heterogeneity. Compared with the traditional linear model, geo-detector was not limited by a linear assumption and could investigate the interactive effects between two independent variables on a dependent variable [61]. The geo-detector contains four main modules, that is, factor detector, risk detector, ecological detector and interaction detector [61]. Given the nonlinear environment-dengue relationship [22, 41], we used factor detector and interaction detector in this study. The monthly dengue cases were used as the dependent variable, and climate and socio-ecological factors were used as the independent variables. Geo-detector requires the independent variable to be a categorical variable [61]. Therefore, climate factors were divided into four classes according to their quartiles. We used the K-means algorithm to classify each socio-ecological factor, so that each factor was changed from a numerical variable to a categorical variable.

Factor detector was used to evaluate explanations of each factor (q(*X*_*i*_)) on dengue according to the following q-statistic [60]:

$$q=1-\frac{\sum_{h=1}^{L}N_{h}\sigma_{h}^{2}}{N\sigma^{2}}=1-\frac{SSW}{SST}$$ (1)

$$SST=N\sigma^{2}$$ (2)

$$SSW=\sum_{h=1}^{L}N_{h}\sigma_{h}^{2}$$ (3)

where h (1, …, L) is the number of strata of environmental factor X; N represents the total number of samples; *σ*^2^ is the variance of dengue cases in the whole study area; *N*_*h*_ means the number of samples in strata h; $\sigma_{h}^{2}$ is the variance in strata h; SSW is the within sum of squares, and SST is the total sum of squares. The calculated q-statistic value ranges from 0 to 1, and the larger q(*X*_*i*_) represents the stronger influence of climate and socio-ecological variable X on dengue case.

Interaction detector can be used to test the interactive effects of two different factors, e.g., *X*_1_ and *X*_2_. The q(*X*_1_) and q(*X*_2_) of factor *X*_1_ and *X*_2_ were calculated from the above factor detector. By overlaying the factor strata *X*_1_ and *X*_2_, we can obtain the interaction q-statistic value written as q (*X*_1_ ∩ *X*_2_). There are five categories of the interactive results (Table 1).

**Table 1:** Types of interactions between the two covariates.

Criterion	Interaction
q (*X*_1_ ∩ *X*_2_) < Min (q(*X*_1_), q(*X*_2_))	nonlinearly weakened
Min (q(*X*_1_), q(*X*_2_)) < q(*X*_1_ ∩ *X*_2_) < Max(q(*X*_1_), q(*X*_2_))	unilaterally nonlinearly weakened
q(*X*_1_ ∩ *X*_2_) > Max(q(*X*_1_), q(*X*_2_))	bilaterally enhanced
q(*X*_1_ ∩ *X*_2_) = q(*X*_1_) + q(*X*_2_)	independent
q(*X*_1_ ∩ *X*_2_) > q(*X*_1_) + q(*X*_2_)	nonlinearly enhanced

Based on the above analysis, we examined the interactive impacts of the seven key climate and socio-ecological factors on dengue transmission. Then we used earth system model data, evidence and findings from related literature to interpret the future trends of key factors. Combining the effects of the factors on dengue during the outbreak period and the trends of the key factors, we qualitatively assessed the possibility of dengue outbreaks in the future.

## Results

### Effects of climate and socio-ecological factor on dengue incidence over long-time series

**Fig 2:**
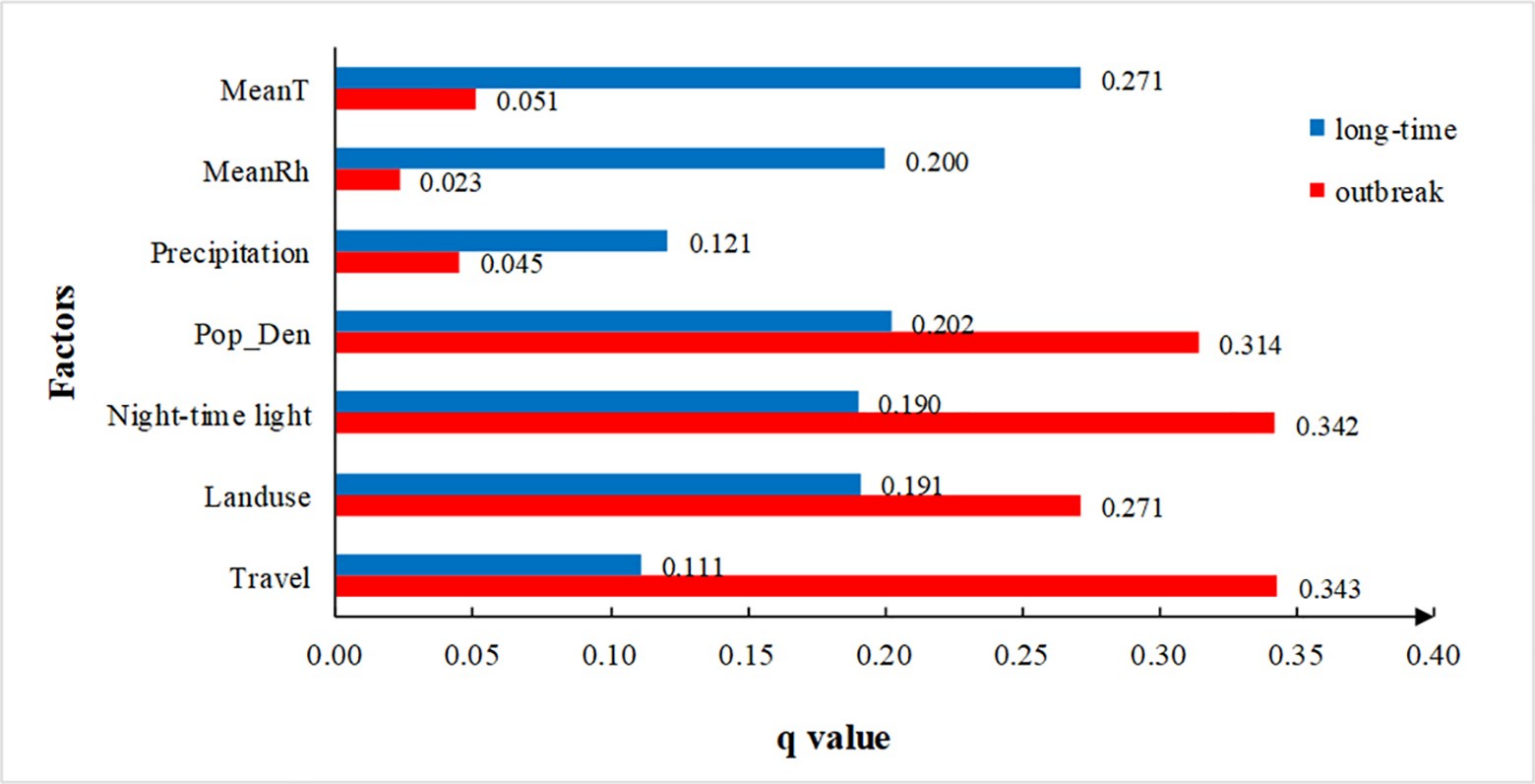
Comparison of the power of determinant (q value) for seven factors affecting dengue transmission in Guangzhou from 1998 to 2014. MeanT: mean temperature; MeanRh: mean relative humidity; Pop_Den: population density; Travel: the number of travelers; Landuse: proportional area of forest, grassland and waters.

The q values of individual factors over the long-time series (blue bars in Fig 2) are as follows: MeanT (0.271), MeanRh (0.200), Precipitation (0.121), Pop_Den (0.202), night-time light (0.190), Landuse (0.191) and Travel (0.111). It indicates that mean temperature, with the highest q value, can predominantly explain dengue incidence followed by population density and mean humidity. Precipitation and the number of travelers are proven to have a weak explanatory influence on dengue incidence. The calculated q values for all factors are between 0.111 and 0.271, thus the differences are not large among factors. This implies that both climate and socio-ecological aspects are important for dengue transmission in Guangzhou over long-term periods. Furthermore, temperature is the dominant driver for dengue incidence, which provides a basis for the seasonality of dengue incidence.

**Fig 3:**
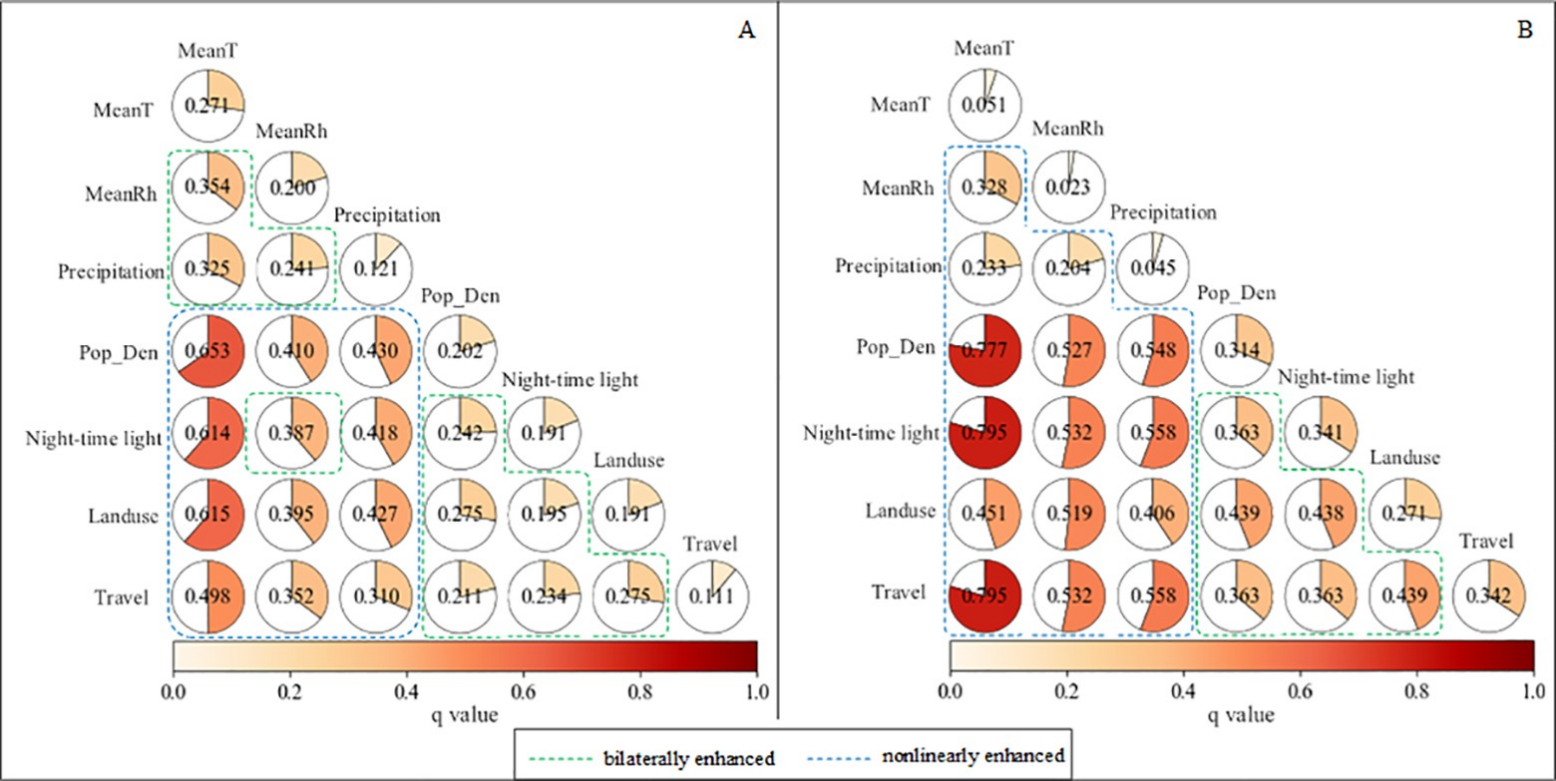
Interactive effects of each paired factors on dengue incidence in Guangzhou from 1998 to 2014. A: long-time series; B: outbreak periods. MeanT: mean temperature; MeanRh: mean relative humidity; Pop_Den: population density; Travel: the number of travelers; Landuse: proportional area of forest, grassland and waters.

The interactive effects of climate and socio-ecological factors on dengue incidence over the long-time series are shown in Fig 3A. First, the explanatory power (q (*X*_1_ ∩ *X*_2_)) of any two independent factors has been enhanced after interaction, either bilaterally or nonlinearly. This indicates that the interaction between two factors contributes more to dengue incidence than that by a single factor. That is, the effects of these seven climate and socio-ecological factors together on dengue are mutually promoting.

Second, the interaction among the same type of factors is weaker than that among different types of factors. The effects of climate factors including MeanT, MeanRh and Precipitation on dengue incidence are mutually enhanced, but this enhancement is not strong. For example, the q values of MeanT and MeanRh are 0.271 and 0.200 respectively, and the q value of their interaction reaches 0.354 (Max(q(MeanT), q(MeanRh)) < q(MeanT ∩ MeanRh) < q(MeanT)+q(MeanRh)). This suggests that, although temperature and humidity have a significant impact on dengue incidence, this effect is limited with increasing temperature and humidity. Similar enhancement occurs between socio-ecological factors including Pop_Den, night-time light, Landuse and Travel. However, the interactive effects of climate and socio-ecological factors on dengue incidence are mostly nonlinearly enhanced (blue boxes in Fig 3A), except that the effect of MeanRh and night-time light is bilaterally enhanced.

Third, among the pairs of climate and socio-ecological factors, the dominant interactive power q (*X*_1_ ∩ *X*_2_) is MeanT that interacts with Pop_den, night-time light, Landuse and Travel. Their q values are 0.653, 0.614, 0.615 and 0.498, respectively. This indicates that the interactive effect of temperature and socio-ecological factors on dengue incidence is evidently stronger, compared to that of precipitation and humidity. Therefore, temperature also plays an important role in the interaction effect.

### Effects of climate and socio-ecological factor on dengue incidence in outbreak periods

Explanatory power (q) of climate and socio-ecological factors on dengue incidence in outbreak periods is shown in Fig 2, expressed as red bars. The q value of each factor is ranked as follows: Travel > night-time light > Pop_den > Landuse > MeanT > Precipitation >MeanRh. The q values for all factors are between 0.023 and 0.343. This range indicates that the difference is large between factors at a given time period, compared with the results over long-term periods (blue bars in Fig 2). The dominant power of socio-ecological factors is much greater than that of climate factors. This indicates that socio-ecological factors significantly contribute to rapid transmission of dengue and, with high incidence, may lead to dengue outbreaks.

Fig 3B shows the interactive effect of climate and socio-ecological factors on dengue incidence in outbreak periods. First, the interactive effect of any two factors is similarly greater than the contribution of a single factor to dengue incidence. For example, the q value reaches 0.328 (q(MeanT ∩ MeanRh)) after interaction of mean temperature and mean humidity, which is far higher than their separate influence (q(MeanT = 0.051 and q(MeanRh) = 0.023) on dengue incidence.

Second, different from the results over long-time series (Fig 3A), the interactions of all pairs of climate factors and socio-ecological factors are all nonlinearly enhanced, with higher q values (blue boxes in Fig 3B). This indicates that this combination could greatly promote dengue incidence in comparison with either the pairs of climate factors or the pairs of socio-ecological factors. For instance, the q value of MeanRh is 0.023, and the q value of Pop_Den is 0.314. After their interaction, the q value reaches 0.527 (q(MeanRh ∩ Pop_Den) > q(MeanRh)+q(Pop_Den)). The q value of night-time light is 0.341, and the q value of interaction of night-time light and Pop_Den is 0.363 (Max(q(Pop_Den), q(night-time light)) < q(Pop_Den ∩ night-time light) < q(Pop_Den)+q(night-time light)).

Third, compared with other climate factors, the interactive effects of mean temperature and socio-ecological factors are stronger in outbreak periods. For example, the interaction of mean temperature and the number of travelers can explain nearly 80% of the dengue incidence, with the highest p value. It indicates that temperature also plays a great role in the interaction effect in outbreak periods. The dominant interactive power for dengue is mean temperature interacting with population density, night-time light or the number of travelers, with the higher q values of 0.777, 0.795 and 0.795, respectively. Therefore, mean temperature, population density, night-time light and the number of travelers are the most important factors for dengue, and their combined interaction is likely to cause an outbreak of dengue.

Based on the above two-fold analysis, it is explicitly clear that temperature is a major driver for long-time series of dengue transmission; and mean temperature, population density, night-time light and the number of travelers are the most important factors for dengue outbreaks.

### Assessment of dengue outbreaks

**Fig 4:**
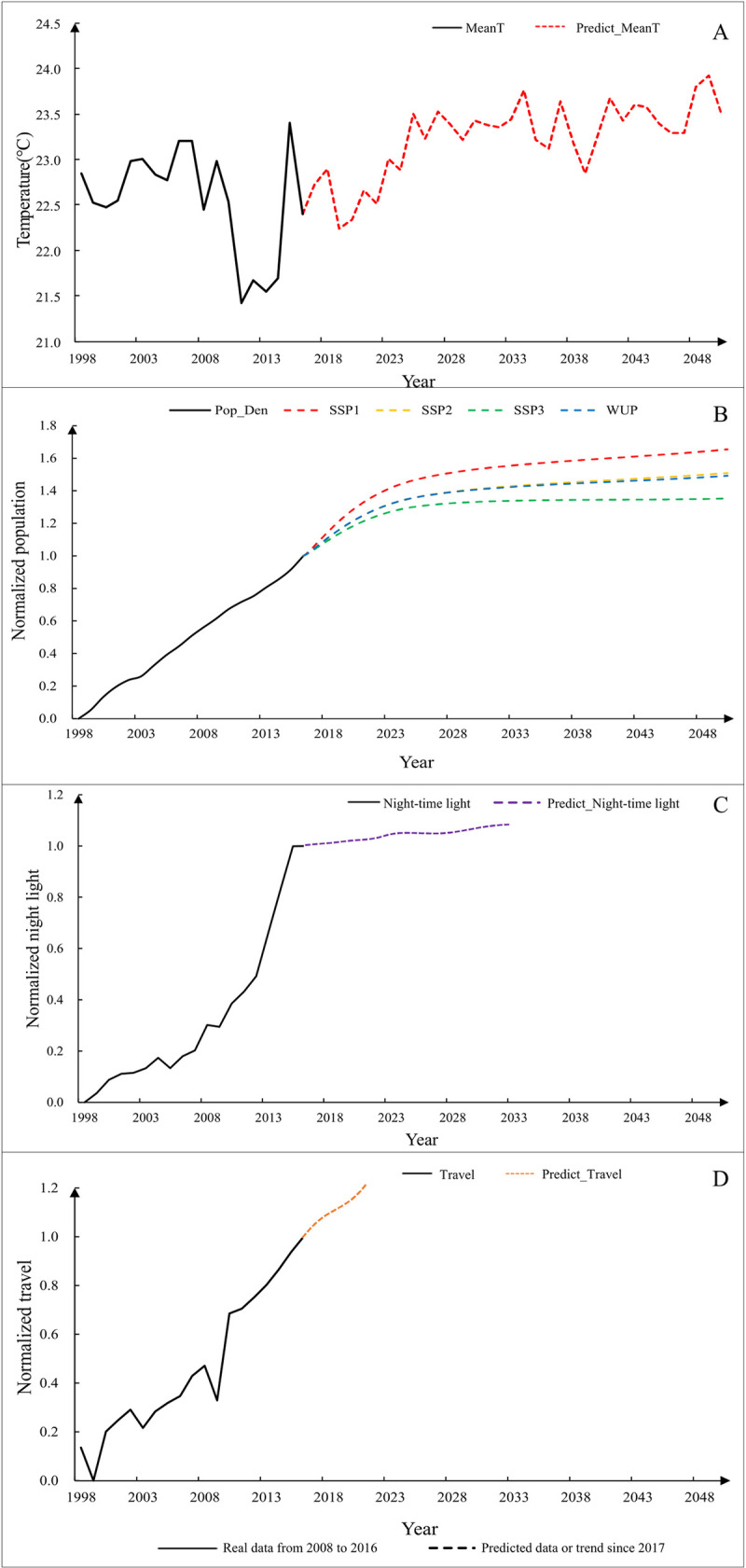
The time-series trends of key factors for dengue outbreaks in Guangzhou since 1998. A: mean temperature (MeanT); B: population density (Pop_Den); C: night-time light; D: the number of travelers (Travel).

Fig 4 displays the time-series trends of four key factors since 1998 in Guangzhou. Fig 4A shows the trend of mean temperature from 1998 to 2050. The solid black line represents the temperature monitored by the weather station from 1998 to 2016. The red dotted line shows the predicted temperature for 2017–2050. Time series data of temperature exhibits an obviously upward trend, which indicates a warming climate condition in the future. Such a climatic environment will favor the survival as well as replication of the dengue virus and the reproduction of *Aedes* mosquitoes, thereby contributing to the future outbreak of dengue.

Fig 4B shows the trend of population density from 1998 to 2050. The solid black line represents the real population density during 1998 and 2016, while the red, yellow, green and blue dotted lines present the predicted population densities for 2017–2050 under the four scenarios. The predicted population density is largest under the scenarios of SSP1, followed by those of SSP2, WUP and SSP3. Overall, the predicted trends of population density under the four scenarios are similar, which grow slowly and then stay flat. Although a high-density population may prompt dengue transmission [62], slow-growing and steady population trends and the population management policies implemented from 2005 in Guangzhou [43] can reduce the likelihood of dengue outbreaks.

Fig 4C shows the time-series trends of the night-time light since 1998. The real data during 1998–2016 and the future trend from 2017 are described by solid black line and red dotted line, respectively. These trend lines indicate that, night-time light rose sharply, which demonstrates that Guangzhou has experienced rapid urbanization during this period. Fang et al. [55] proposed a four-stage theory of China’s urbanization development: an initial stage (when urbanization level is less than 30%), an intermediate stage (when urbanization level is between 30% -60%), a later stage (when urbanization level is between 60% -80%) and a final stage (when urbanization level is higher than 80%). A study used data from 2000 to 2015 to analyze the evolutionary characteristics of urbanization level of Guangzhou. It has found that Guangzhou is in the later or final stage nowadays [56]. Given this, Guangzhou’s urbanization process (reflected as night-time light) can be assumed to grow slowly and stabilize in the future.

Fig 4D shows the long-term trends of the number of travelers since 1998. The solid black line depicts the observed data during 1998–2016, which indicates the number of travelers in Guangzhou continues to surge during this period. A study conducted by the Guangzhou Development Research Institute in 2017 reported that the government would strongly support tourism, and the number of travelers in Guangzhou would increase in the future [57].

According to the predicted future trends of the four indicative factors, both the temperature and travel are determinant predictors for future dengue outbreaks in Guangzhou. More specifically, warmer temperature and increasing travelers are the most likely to cause future dengue outbreaks.

## Discussion and conclusion

In recent years, dengue has posed a serious public health burden in Guangzhou. This study quantitatively and systematically analyzes the relationship between climate as well as socio-ecological factors and dengue transmission during 1998–2014. Our results highlight that the temperature plays a dominant role in the long-term trend of dengue incidence, while socio-ecological factors have great explanatory power for dengue outbreaks. The interactions between pairs of climate and socio-ecological factors have a more significant impact on dengue than any single factor has. According to future trends of the four key factors, increasing temperature and a surge in travel could cause dengue outbreaks in the future.

Temperature plays a great role in the long-term transmission of dengue. This finding is consistent with those from existing studies [14, 31, 39, 63]. Because dengue is affected by the transmission mechanism of "virus-mosquito-vulnerable people", temperature could affect the dengue transmission from the three aspects. First, replication of dengue virus and its extrinsic incubation period are related to temperature. Suitable temperature helps dengue virus survive and shortens their replication time, which promotes dengue transmission [63]. Second, temperature may influence *Aedes* mosquitoes in many ways, including but not limited to their life cycle, biting behavior, density and flying distance [11]. For example, a warmer environment will allow mosquitoes to end the diapause period early in spring and postpone their extinction period in winter, which increases the number and density of mosquitoes throughout the year, thereby increasing the incidence of dengue [64]. Third, temperature also affects people’s behaviors and habits. A warm environment will encourage people to do more outdoor activities, which will increase the possibility of being bitten by mosquitoes with dengue virus.

Socio-ecological factors including population density, night-time light and travel have a more pronounced effect on dengue outbreaks. Our results are also in agreement with many previous studies [65, 66]. For example, a study analyzed the viral genetic information and data on travelers from dengue-endemic Asian countries, and suggested the dengue virus causing the 2014 outbreak in Tokyo, Japan, was likely imported from Guangzhou, China, because of human mobility [66]. Population density reflects the gathering and exposure of people, and is also related to urban expansion and urbanization. These are important aspects closely related to the incidence of dengue. The night-time light reflects the process of urbanization, which affects dengue in Guangzhou. Early rapid urbanization produces many rural-urban fringes, where many people would live with poor housing conditions. This type of living conditions provides suitable breeding sites for *Aedes* mosquitoes and cause dengue transmission. Furthermore, planned urbanization in the later and final stages would have better medical facilities and environmental management [67]. Better medical facilities can provide rapid diagnostic test for patients to prevent further transmission of dengue [68]. Effective environment management, such as timely cleanup of water bodies and garbage in populous places, will reduce mosquito breeding sites [43]. Both of the above will decrease the dengue incidence. In addition, travel also has an important impact on dengue by introducing imported cases. A previous study analyzing the trend of tourism in Guangdong Province indicated that there is an increasing number of inbound travelers [69]. As the capital city of Guangdong Province, the number of inbound travelers would similarly increase in Guangzhou. Such travel is likely to introduce imported dengue cases, especially from endemic regions, and increase the local risk of dengue transmission [70].

Dengue transmission becomes complex due to interaction of multiple factors. Our findings imply that the interaction of temperature and the number of travelers has strongly contributed to the outbreak of dengue in Guangzhou. In China, dengue is recognized as an imported infectious disease [38]. Travel is partly responsible for dengue transmission in two ways: vulnerable people from outside regions could be infected when traveling in epidemic areas; and infected people may bring the dengue virus from epidemic areas to their destination region [14]. Certain environmental conditions induce infections when the dengue virus is present. Therefore, the increase in travelers and the warming local temperatures, which are very favorable for the survival of the virus and the breeding and biting of *Aedes* mosquitoes, may cause future dengue outbreaks. Therefore, if these two aspects are managed and controlled well, there is less chance of dengue outbreaks in Guangzhou in the future.

Based on this study, we propose three recommendations regarding the prevention of dengue outbreaks in Guangzhou. First, mitigating the urban heat island effect to lower temperatures in urban areas is necessary. For example, increasing vegetation cover would be beneficial to decrease the temperature in urban areas. Second, adjusting the time and frequency of vector control intervention is crucial to the prevention and control of dengue. Due to warmer temperatures, mosquitoes will come out diapause earlier [64]. Therefore, the first vector control intervention should be advanced in late spring. And in the high incidence period of summer and autumn, the government should increase the frequency and intensity of mosquito control. Third, providing targeted publicity information to travelers at border points should be considered. At border points, televisions or other electronic display screens can be used to educate travelers from or to areas with high dengue incidence. The recurring information could include dengue symptoms, disease risks, the current pandemic areas of dengue, where and how to seek medical care for the suspected, household inspection for mosquito breeding sites, and personal prevention and control measures.

This study presents improvements in two aspects. It is conceived that vector-borne diseases analyses should incorporate independent and interacting effects of global change. But such analysis on dengue is lacking. The current study investigates independent and interacting effects of climate and socio-ecological factors on dengue incidence from two perspectives: long-time series and outbreak periods. This is a comprehensive study to help us understand the multi-factor and complex process of dengue transmission. In addition, we assess the possibility of dengue outbreaks in Guangzhou based on the future trends of high-risk factors. This is the first study to analyze the future possibility of dengue outbreaks based on both climate and socio-ecological factors.

However, the limitations of this study should also be acknowledged. For example, other factors such as mosquito density, human immunity and human intervention, were not considered in this study due to unavailability of data. They are also known key drivers of dengue transmission, and likely influence outbreaks studied herein. In addition, we currently do not obtain finer spatial-scale and updated dengue case data, so we cannot conduct a recent study and analyze the spatial heterogeneity of dengue incidence. Considering the spatiotemporal analysis of dengue transmission will produce more accurate results and provide more targeted prevention and control recommendations. Nevertheless, analysis derived from this study can help us to better understand how climate and socio-ecological factors might affect dengue transmission, and how to control and prevent dengue outbreaks.
